# *Pseudomonas aeruginosa* Protease IV Exacerbates Pneumococcal Pneumonia and Systemic Disease

**DOI:** 10.1128/mSphere.00212-18

**Published:** 2018-05-02

**Authors:** Jessica L. Bradshaw, Armando R. Caballero, Michael A. Bierdeman, Kristen V. Adams, Haley R. Pipkins, Aihua Tang, Richard J. O’Callaghan, Larry S. McDaniel

**Affiliations:** a Department of Microbiology and Immunology, University of Mississippi Medical Center, Jackson, Mississippi, USA; b Department of Pathology, University of Mississippi Medical Center, Jackson, Mississippi, USA; University of Kentucky

**Keywords:** Pseudomonas aeruginosa, Streptococcus pneumoniae, coinfection, invasive disease, pneumococcus, pneumonia, proteases

## Abstract

Pneumonia is a pulmonary disease affecting people of all ages and is consistently a leading cause of childhood mortality and adult hospitalizations. Streptococcus pneumoniae and Pseudomonas aeruginosa are major lung pathogens commonly associated with community-acquired and nosocomial pneumonia. Additionally, mixed lung infections involving these bacterial pathogens are increasing in prevalence and are frequently more severe than single infections. The cooperative interactions of these two pathogens that impact pulmonary disease severity are understudied. A major secreted virulence factor of P. aeruginosa, protease IV (PIV), cleaves interleukin 22 (IL-22), a cytokine essential for maintaining innate mucosal defenses against extracellular pathogens. Here, we investigate the ability of PIV to augment the virulence of a pneumococcal strain with limited virulence, S. pneumoniae EF3030, in a C57BL/6 murine model of pneumonia. We demonstrate that pulmonary coinfection involving P. aeruginosa 103-29 and S. pneumoniae EF3030 results in pneumococcal bacteremia that is abrogated during pneumococcal coinfection with a PIV-deficient strain. Furthermore, intratracheal administration of exogenous PIV and EF3030 resulted in abundant immune cell infiltration into the lung with large abscess formation, as well as severe bacteremia leading to 100% mortality. Heat-inactivated PIV did not worsen pneumonia or reliably induce bacteremia, suggesting that the specific activity of PIV is required. Our studies also show that PIV depletes IL-22 *in vivo*. Moreover, PIV-mediated enhancement of pneumonia and disease severity was dependent on the expression of pneumolysin (Ply), a prominent virulence factor of S. pneumoniae. Altogether, we reveal that PIV and Ply additively potentiate pneumonia in a murine model of lung infection.

**IMPORTANCE** S. pneumoniae remains the leading cause of bacterial pneumonia despite widespread use of pneumococcal vaccines, forcing the necessity for appropriate treatment to control pneumococcal infections. Coinfections involving S. pneumoniae with other bacterial pathogens threaten antibiotic treatment strategies and disease outcomes. Currently, there is not an effective treatment for alveolar-capillary barrier dysfunction that precedes bacteremia. An understanding of the dynamics of host-pathogen interactions during single and mixed pulmonary infections could elucidate proper treatment strategies needed to prevent or reduce invasive disease. Antibiotic treatment decreases bacterial burden in the lung but also increases acute pathology due to cytotoxins released via antibiotic-induced bacterial lysis. Therefore, targeted therapeutics that inhibit or counteract the effects of bacterial proteases and toxins are needed in order to limit pathology and disease progression. This study identifies the cooperative effect of PIV and Ply, products of separate lung pathogens that additively alter the lung environment and facilitate invasive disease.

## Abstract (precis)

S. pneumoniae remains the leading cause of bacterial pneumonia despite widespread use of pneumococcal vaccines, forcing the necessity for appropriate treatment to control pneumococcal infections. Coinfections involving S. pneumoniae with other bacterial pathogens threaten antibiotic treatment strategies and disease outcomes. Currently, there is not an effective treatment for alveolar-capillary barrier dysfunction that precedes bacteremia. An understanding of the dynamics of host-pathogen interactions during single and mixed pulmonary infections could elucidate proper treatment strategies needed to prevent or reduce invasive disease. Antibiotic treatment decreases bacterial burden in the lung but also increases acute pathology due to cytotoxins released via antibiotic-induced bacterial lysis. Therefore, targeted therapeutics that inhibit or counteract the effects of bacterial proteases and toxins are needed in order to limit pathology and disease progression. This study identifies the cooperative effect of PIV and Ply, products of separate lung pathogens that additively alter the lung environment and facilitate invasive disease.

## INTRODUCTION

Pneumonia is the leading cause of childhood mortality worldwide and is also a primary reason for hospitalizations associated with high morbidity and financial burden in the United States (1, 2). The causative agent in cases of pneumonia has classically been diagnosed and treated as a single infecting microorganism, and the principal etiological agents identified during episodes of bacterial pneumonia include Streptococcus pneumoniae, Haemophilus influenzae, Staphylococcus aureus, and Pseudomonas aeruginosa (3). However, increases in concurrent lung infections have been reported during both nosocomial and community-acquired pneumonia (CAP), and these mixed bacterial infections have resulted in more severe disease presentations than single infections (4–8). Specifically, S. pneumoniae is the most common causative agent of CAP and is also the most prevalent bacterium during mixed CAP infections (5–7).

In healthy individuals, the environment of the lower respiratory system is nutrient poor and unfavorable to invading pathogens (9). During acute lung injury or chronic lung disease, the alveolar environment favors bacterial growth through nutrient-rich fluid accumulation, temperature gradients, development of oxygen pockets, and a host inflammatory response that selectively favors potential pathogens (10–13). Contrary to the previous assumption that the lung is a sterile environment, a heterogenous microbiome has been identified in the lungs of healthy individuals (9). Furthermore, the lung microbiome shapes the ecological forces that influence the colonization or elimination of potential pathogens in the lower airways (10). Lung microbiome dysbiosis occurs during acute lung injury such as viral infection and favors the growth of P. aeruginosa and S. pneumoniae (10). Moreover, lung coinfections linking P. aeruginosa and S. pneumoniae have been reported (7, 14–16). This coexistence implies that these two pathogens are cooperatively interacting during pneumonia. These interactions may be a result of secreted virulence factors that alter the lung environment, select for outgrowth of these pathogens, and thus enhance persistence of both pathogens.

Here, we investigated the ability of a secreted *Pseudomonas* protease, protease IV (PIV), to augment an active lung infection caused by S. pneumoniae EF3030, a strain of pneumococcus with limited virulence in a murine model (17). PIV has been shown to alter the lung mucosal defense by proteolytically degrading host immune effectors, such as interleukin 22 (IL-22) and surfactant proteins (18, 19). IL-22 is a critical facilitator of mucosal innate immunity whose effects maintain mucosal barrier integrity through specific targeting of mucosal epithelial cells to induce tissue repair and secretion of antimicrobial peptides (20). Surfactant proteins are innate immune effectors that bind, aggregate, and increase phagocytic uptake of bacterial pathogens in the lung (21). Furthermore, IL-22 and surfactant proteins have been shown to be essential for limiting pneumococcal pneumonia, and EF3030 is a pneumococcal strain that does not readily induce bacteremia (22, 23). Thus, a lung environment altered by PIV activity may favor the outgrowth of pneumococci, leading to enhanced lung pathology driven by pneumococcal virulence factors. Pneumolysin (Ply) is a cytotoxin and prominent virulence factor of pneumococci that has been shown to be essential during pulmonary infection and dissemination into the bloodstream (24–27). Based on these prior studies, we hypothesize that depletion of IL-22 by PIV in the localized lung environment exacerbates pneumococcal pneumonia and permits invasive disease mediated by Ply.

## RESULTS

### Pulmonary coinfection involving S. pneumoniae and P. aeruginosa expressing PIV results in pneumococcal bacteremia.

**FIG 1:**
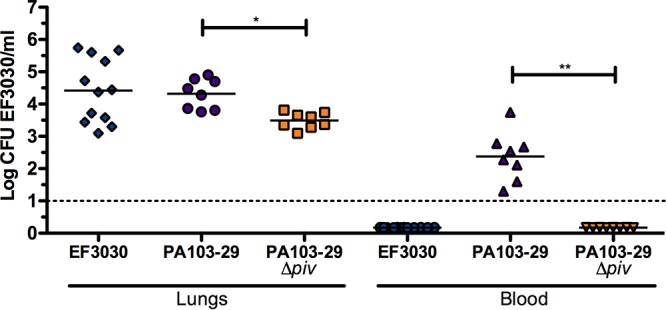
P. aeruginosa expressing protease IV (PIV) during murine lung coinfection with S. pneumoniae EF3030 stimulates pneumococcal bacteremia. C57BL/6 mice were intratracheally inoculated with 10^3^ CFU of P. aeruginosa 103-29 (PA103-29) or PA103-29 *piv* deletion mutant followed by intratracheal inoculation of 10^6^ CFU of S. pneumoniae EF3030 at 24 h after *Pseudomonas* infection. Pneumococcal burden in the lungs and blood was enumerated by plating on selective blood agar 48 h after pneumococcal infection. Deletion of *piv* in PA103-29 significantly reduced pneumococcal load in the lung and abrogated pneumococcal bacteremia. Each symbol represents the value for a single mouse, and each bar represents the mean for that group of mice. Data are representative of two independent studies. *, *P* < 0.05; **, *P* < 0.01.

EF3030 is a pneumococcal strain with limited virulence that does not readily induce bacteremia during pneumonia in a murine model of infection. We performed a coinfection of S. pneumoniae EF3030 with either the P. aeruginosa 103-29 parent strain (designated PA103-29) or a *piv* deletion mutant of PA103-29 to determine if secreted virulence factors of P. aeruginosa could stimulate pneumococcal bacteremia. In mice coinfected with EF3030 and PA103-29, there was no significant difference in pneumococcal densities in the lungs compared to mice infected with EF3030 alone (Fig. 1). However, pneumococci were isolated from the blood of all mice coinfected with EF3030 and PA103-29. A deletion of *piv* in PA103-29 significantly reduced (*P* < 0.001) pneumococcal loads in murine lungs and abrogated pneumococcal bacteremia (Fig. 1).

### Protease IV is active in murine lungs during infection.

**FIG 2:**
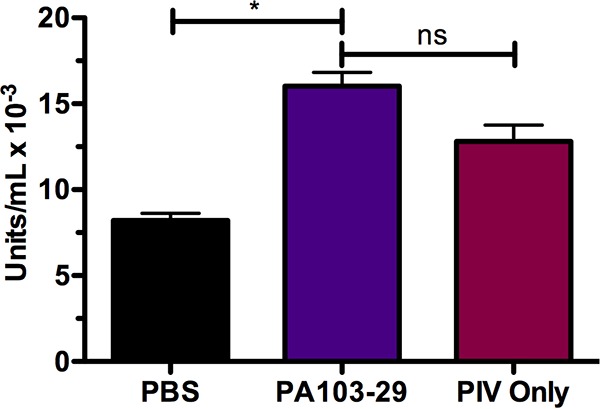
Protease activity in murine lungs at 24 h after intratracheal administration. C57BL/6 mice were intratracheally inoculated with phosphate-buffered saline (PBS; control), 10^6^ CFU of P. aeruginosa 103-29 (PA103-29), or 10 µg protease IV (PIV). At 24 h after intratracheal administration, protease activity in murine lungs was measured on a spectrophotometer using the chromogenic substrate Chromozym PL. Optical density readings at 405 nm were converted into proteolytic units per milliliter as described by the manufacturer. Protease activity significantly increased during PA103-29 infection, and there was no significant difference between protease activity measured during active infection and that measured during administration of PIV alone. Data represent two independent studies performed in triplicate. Error bars represent standard errors of the means. *, *P* < 0.05; ns, no significant difference.

To further examine PIV-specific effects on pneumococcal pulmonary infections, we measured protease activity in lung homogenates of mice intratracheally administered phosphate-buffered saline (PBS), PA103-29, or PIV alone to determine a physiologically relevant concentration of PIV to administer (Fig. 2). When evaluating the secretome of P. aeruginosa, the Chromozym PL assay has been previously shown to be specific for measuring PIV activity (28). Compared to mock-treated mice, significantly higher (*P* < 0.05) proteolytic activity was measured in the lungs of mice during PA103-29 infection. Administration of 10 µg PIV resulted in a nonsignificant 0.25-fold decrease in proteolytic activity compared to proteolytic activity in the lungs of mice during active infection (Fig. 2).

### PIV exacerbates pneumococcal pneumonia that develops into highly fatal bacteremia in a murine model of infection.

**FIG 3:**
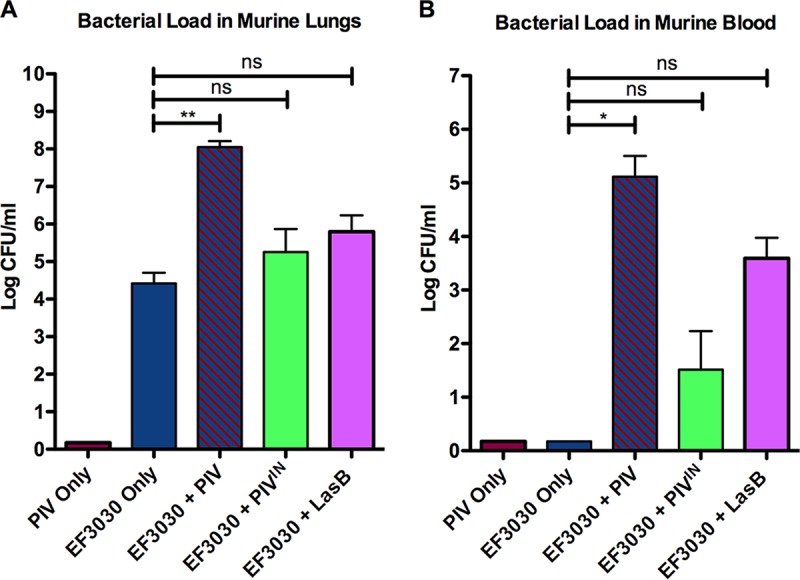
*Pseudomonas* PIV enhances pneumococcal pneumonia and bacteremia in C57BL/6 mice. Mice were intratracheally inoculated with 10 µg of *Pseudomonas* PIV, 10^6^ CFU of pneumococci, or mixtures of 10^6^ CFU pneumococci with 10 µg of respective *Pseudomonas* proteases. Pneumococcal burdens in the lungs (A) and blood (B) of mice were determined 2 days after lung infection. PIV^IN^, heat-inactivated PIV. PIV significantly increased pneumococcal burden in the murine lungs (A) and led to bacteremia (B). Data shown are representative of two independent experiments (*n* ≥ 6). Error bars represent standard errors of the means. *, *P* < 0.05; **, *P* < 0.01; ns, no significant difference.

We investigated whether the proteolytic effects of PIV could augment the virulence of EF3030 in a C57BL/6 murine pneumonia model. LasB is a *Pseudomonas* protease that cleaves a number of host proteins, including surfactants, but this specific protease does not cleave IL-22 (18). Therefore, LasB was used as a negative control for IL-22 cleavage. EF3030 combined with PIV significantly increased (*P* < 0.01) bacterial burden during pneumococcal pneumonia (Fig. 3A) and allowed the infection to advance to significant bacteremia (*P* < 0.05) compared to EF3030 alone (Fig. 3B). Heat-inactivated PIV or LasB did not significantly increase bacterial burden during pneumococcal pneumonia compared to EF3030 alone (Fig. 3A). However, there was a strong trend (*P* = 0.0625) toward increased bacteremia in mice inoculated with LasB compared to EF3030 alone (Fig. 3B). PIV and EF3030 combined inoculation resulted in 100% mortality, while all other inoculations caused very low or no mortality by 2 days postinfection (see Fig. 5).

### PIV increases host susceptibility to Ply.

**FIG 4:**
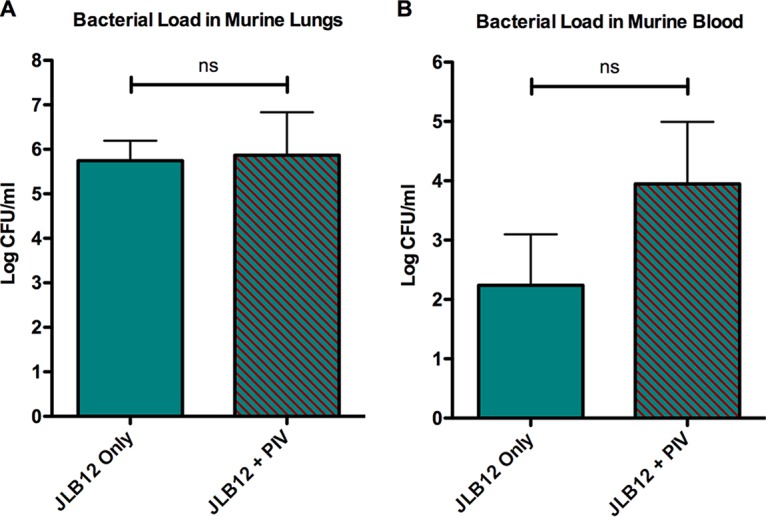
Pneumolysin (Ply) is required for PIV-mediated enhancement of pneumococcal pneumonia in a murine model. Mice were intratracheally inoculated with 10^6^ CFU of an EF3030 *ply* mutant (JLB12) with or without 10 µg of *Pseudomonas* PIV. Mouse lungs and blood were collected 2 days following lung infection, and pneumococcal CFU were enumerated by plating on BA. PIV did not significantly enhance pneumococcal burden in the lungs (A) or blood (B) of mice infected with a pneumolysin-deficient mutant. Data shown are representative of two independent studies (*n* ≥ 10). Error bars represent standard errors of the means. ns, no significant difference.

**FIG 5:**
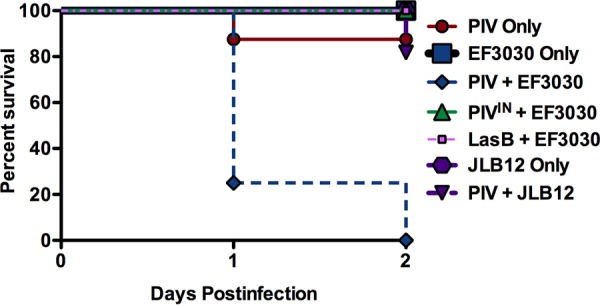
*Pseudomonas* PIV and pneumococcal pneumolysin contribute to murine mortality during experimental pneumonia. Murine survival was monitored for 2 days following pulmonary infection. Survival rates are expressed as percentages (PIV, *n* = 8; EF3030, *n* = 12; EF3030 + PIV, *n* = 8; EF3030 + PIV^IN^, *n* = 10; EF3030 + LasB, *n* = 6; JLB12, *n* = 10; JLB12 + PIV, *n* = 11). Mice infected simultaneously with *Pseudomonas* PIV and S. pneumoniae EF3030 expressing pneumolysin experienced 100% mortality, while all other inoculations resulted in very little to no mortality by 2 days postinfection. Data are representative of two independent experiments. Survival curves were determined to be significantly different (*P* < 0.0001) by log rank (Mantel-Cox) test.

Pneumolysin (Ply) is a virulence factor of pneumococcus that compromises alveolar-capillary barrier function, leading to permeability and flooding of alveoli (29). Since PIV enhanced pneumococcal pneumonia in a murine model (Fig. 3), we examined whether this increase in pathology was a result of additive Ply activity. Whether PIV was administered or not, there was no significant difference in bacterial load in the lungs (Fig. 4A) or blood (Fig. 4B) of mice during experimental pneumonia when infected with the Ply-deficient strain JLB12. Higher CFU were recovered from mice infected with JLB12 alone (Fig. 4A) than from mice infected with EF3030 alone (Fig. 3A), with some mice infected with JLB12 alone becoming bacteremic (Fig. 4B). Nonetheless, all mice infected with either EF3030 or JLB12 alone survived through 2 days postinfection (Fig. 5). Moreover, PIV administered with EF3030 resulted in higher bacterial burdens in murine lungs and blood (Fig. 3) as well as significantly increased mortality (Fig. 5) compared to combined PIV and JLB12-infected mice (Fig. 4 and 5).

### PIV does not impact pneumococcal adherence to human pulmonary epithelial cells.

**FIG 6:**
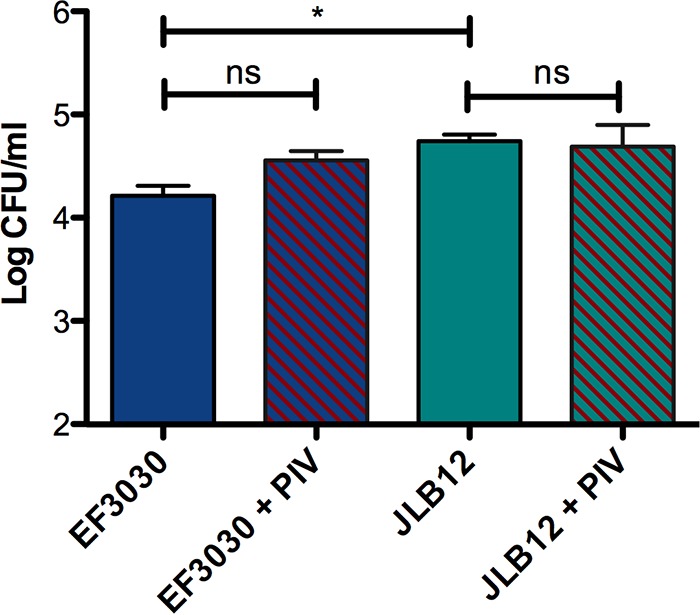
*Pseudomonas* protease IV (PIV) does not alter pneumococcal adherence to human pulmonary epithelial cells. Pneumococci (10^6^ CFU) with or without 10 µg PIV were incubated with A549 pulmonary epithelial cells, and S. pneumoniae adherence to epithelial cells was assessed by plating on BA. EF3030 is an encapsulated serotype 19F strain, and JLB12 is an EF3030 isogenic pneumolysin (*ply*) deletion mutant. PIV did not alter adherence of EF3030 or the *ply* deletion mutant. Deletion of *ply* in EF3030 (JLB12) significantly increased adherence to pulmonary epithelial cells. Data represent two independent studies performed in triplicate. Error bars represent standard errors of the means. *, *P* < 0.05; ns, no significant difference.

Pneumococcal adherence to human pulmonary epithelial cells was examined to determine if PIV alters S. pneumoniae EF3030 interactions with cells relevant to bacterial pneumonia. PIV coadministered with bacterial cells did not impact adherence to epithelial cells in either EF3030 or JLB12, a *ply* mutant strain of EF3030 (Fig. 6). However, a deletion of *ply* significantly increased (*P* < 0.05) pneumococcal adherence to pulmonary epithelial cells (Fig. 6). EF3030 and JLB12 were not able to invade epithelial cells with or without the presence of PIV (data not shown).

### PIV and Ply induce neutrophil (polymorphonuclear leukocyte [PMN]) recruitment and lung damage during active pneumonia.

**FIG 7:**
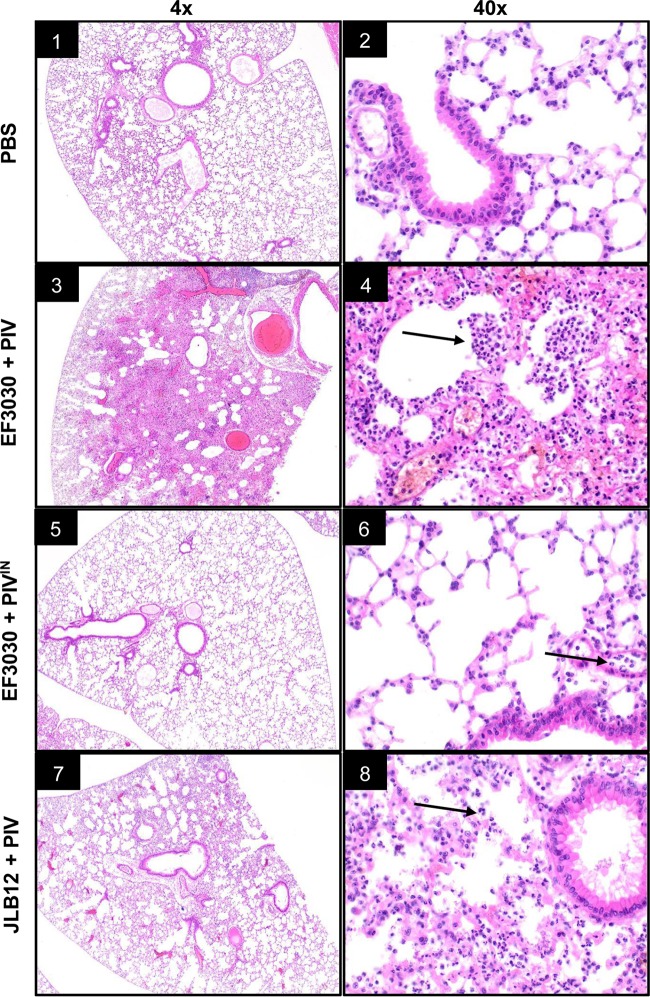
P. aeruginosa PIV and S. pneumoniae producing pneumolysin induce massive neutrophil recruitment and lung damage during active murine pneumonia. Histological analysis of murine lung sections at magnifications of ×4 (left) and ×40 (right). Data are representative of two biological replicates of mice inoculated with PBS (panels 1 and 2), S. pneumoniae EF3030 and PIV (panels 3 and 4), EF3030 and heat-inactivated PIV (PIV^IN^; panels 5 and 6), and JLB12 (EF3030 Δ*ply*) and PIV (panels 7 and 8). EF3030 and PIV combined inoculation resulted in severe, diffuse pneumonia (panels 3 and 4) compared to restricted, focal pneumonia during JLB12 infection (panels 7 and 8). Preserved airspaces and healthy tissues were observed in the inactivated PIV (panels 5 and 6)- and PBS (panels 1 and 2)-treated mice. Arrows indicate areas of acute inflammatory cells within the lung parenchyma, with the largest abscess formation seen within panel 4.

We used histology to visualize relative lung pathology during active pneumonia. Intratracheal administration of EF3030 and PIV led to massive infiltration of immune cells and shrinkage of alveolar spaces with evidence of necrosis and abscess formation (Fig. 7, panels 3 and 4). No indication of pneumonia was observed when mice were inoculated with PBS or with EF3030 combined with heat-inactivated PIV (Fig. 7, panels 1 and 2 and panels 5 and 6, respectively). Mice inoculated with a combination of active PIV and the Ply-deficient strain JLB12 displayed evidence of focal, mild pneumonia characterized by restricted infiltration of immune cells and attenuated shrinking of alveolar air spaces (Fig. 7, panels 7 and 8).

### PIV depletes IL-22 levels *in vivo* during active pneumococcal pneumonia.

**FIG 8:**
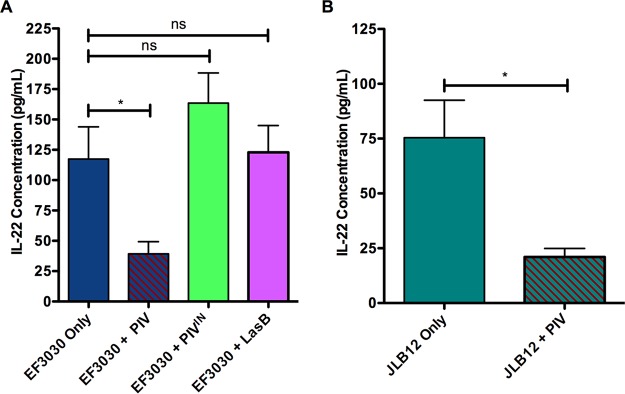
PIV depletes interleukin 22 (IL-22) protein levels *in vivo*. IL-22 was measured in lung homogenates using a sandwich ELISA. Mice infected with active PIV and either EF3030 (A) or pneumolysin-deficient JLB12 (B) had significantly less IL-22 produced during active pneumonia. Error bars represent standard errors of the means. Data are representative of three independent studies performed in triplicate. *, *P* < 0.05; ns, no significant difference.

IL-22 is required for host control of pneumococcal pneumonia, and IL-22 is cleaved by *Pseudomonas* PIV but not LasB (18, 22). To assess if IL-22 cleavage was occurring *in vivo*, we measured IL-22 levels in murine lungs during infection. IL-22 levels were significantly reduced (*P* < 0.05) when PIV was administered in combination with either EF3030 (Fig. 8A) or JLB12 (Fig. 8B) compared to IL-22 levels in mice infected with EF3030 alone or JLB12 alone, respectively. When mice were inoculated with heat-inactivated PIV or LasB, there was no significant difference in IL-22 levels compared to inoculation with EF3030 alone (Fig. 8A). IL-22 levels were also higher in mice infected with EF3030 (Fig. 8A) than in mice infected with the Ply-deficient strain JLB12 (Fig. 8B).

## DISCUSSION

In this study, we demonstrate that a *Pseudomonas* protease augments the virulence of an S. pneumoniae strain in a murine model of acute lung infection. Our findings reveal that PIV and Ply, virulence factors exclusively expressed by two separate pathogens, additively potentiate an active lung infection. The coadministration of active PIV and Ply-expressing strain EF3030 resulted in the highest bacterial loads and severe bacteremia (Fig. 3), as well as 100% mortality (Fig. 5) and enhanced immune cell infiltration leading to large abscess formation (Fig. 7, panel 4). This severe pathology must be attributed to a protease function that is not shared by PIV and LasB, as LasB coadministration with EF3030 did not result in significantly increased pulmonary bacterial loads, bacteremia, or mortality (Fig. 3 and 5). Since PIV did not impact interactions of S. pneumoniae EF3030 with pulmonary epithelial cells (Fig. 6), it is likely that altered immune signaling events are responsible for increased pathology within our murine model of lung infection. Both PIV and LasB cleave host surfactant proteins, but PIV exclusively cleaves IL-22 (18). To our knowledge, the activity of exogenously administered PIV has not been previously investigated in the lung. Our findings were able to quantify PIV activity (Fig. 2) and confirm the depletion of IL-22 by PIV *in vivo* (Fig. 8). This observation during an active murine lung infection is consistent with previous findings of IL-22 degradation in tracheal aspirates of P. aeruginosa-infected patients (18). Therefore, it is likely that IL-22 depletion in the lung is allowing for enhanced pneumococcal burden. PIV is a very weak immunogen (30), so increased pathology was not an effect of PIV immunogenic properties perpetuating deleterious inflammation. Accordingly, specific activity of PIV was required, as heat inactivation of PIV attenuated pathological findings (Fig. 3, 5, and 7). Although IL-22 played a large role in severity of pneumonia in our studies, the role of surfactants cannot be overlooked. LasB coadministration with EF3030 did enhance bacteremia, albeit not to statistical significance (Fig. 3B). Since surfactants have been shown to be important in restricting pneumococcal pneumonia (23), it is likely that the compounded effects of both surfactant and IL-22 depletion by PIV are aiding in pneumococcal ability to overcome mucosal barriers to invasive disease.

Nonetheless, increased pathology was not solely dependent on the activity of PIV. PIV coadministration with Ply-deficient strain JLB12 did not enhance pneumococcal pneumonia or bacteremia (Fig. 4) and resulted in restricted, focal evidence of pneumonia (Fig. 7). Surprisingly, we recovered higher pneumococcal CFU from the lungs of mice infected with JLB12 alone than from mice infected with EF3030 alone (Fig. 3A and 4A), and 50% of mice inoculated with JLB12 alone were bacteremic at 2 days postinfection (Fig. 4B). However, increased adherence to pulmonary cells (Fig. 6) and decreased induction of protective IL-22 (Fig. 8) afforded by JLB12 compared to EF3030 may have allowed for the increased pneumococcal burden in the lung and breakdown of epithelial barrier integrity. The major cellular sources of IL-22 during pneumococcal pneumonia are innate-like cells (ILCs) and thymocyte cells (T cells) expressing γδ T-cell receptors, which are stimulated by IL-23 secreted from activated dendritic cells and macrophages in the lung (22, 31). Ply has been shown to activate macrophages through Toll-like receptor 4 (TLR-4) signaling (32). Thus, decreased activation of macrophages responsible for producing proinflammatory cytokines is likely contributing to the decreased induction of IL-22 and enhanced bacteremia in JLB12-infected mice (Fig. 4B and 8).

Overall, innate mucosal barriers to infection play an important role in preventing pneumococcal invasive disease (PID). Altered lung environments provided by comorbidities or coinfection that lead to microbiome dysbiosis and pathogen outgrowth result in intimate interactions between invading pathogens. Specifically, our study reveals that a secreted *Pseudomonas* virulence factor dampens the host immune response and intensifies disease severity during S. pneumoniae pulmonary infections. Our findings also show that *Pseudomonas* PIV and *Streptococcus* Ply production have a combinatorial effect on disease severity. Unfortunately, the effects of these combined virulence factors cannot be alleviated with antibiotic usage. Since Ply is not secreted and is released upon bacterial lysis (33, 34), bactericidal antibiotics that cause a rapid release of Ply could result in major lung damage. In an altered lung such as the environment created by PIV, increased damage mediated by rapid Ply release could be catastrophic. Thus, studies examining targeted therapeutics that minimize damage created by proteases and cytotoxins are necessary in order to develop proper treatment strategies for patients with critical lung infections.

## MATERIALS AND METHODS

### Materials.

Recombinant P. aeruginosa protease IV (PIV) was purified and assayed using Chromozym PL (Roche) as previously described (35). PIV was heat inactivated at 60°C for 1 h, and inactivity was verified by gelatinase zymography. P. aeruginosa protease LasB was purchased from Elastin Products Company, Inc. Human pulmonary epithelial cell line A549 (ATCC CCL-185) was obtained from Stephen Stray (University of Mississippi Medical Center, Jackson, MS) and authenticated by the American Type Culture Collection on 23 October 2017. Mouse IL-22 Ready-Set-Go! was purchased from Invitrogen. C57BL/6 (B6) mice were bred at the University of Mississippi Medical Center or purchased from The Jackson Laboratory.

### Bacterial strains and growth conditions.

S. pneumoniae strains were grown at 37°C with 5% CO_2_ in Todd-Hewitt broth supplemented with 0.5% yeast extract (THY) or on sheep blood agar (BA) containing 0.5 µg/ml gentamicin. EF3030 is a serotype 19F S. pneumoniae strain (36). An EF3030 isogenic pneumolysin (*ply*) deletion mutant, JLB12, was created by allelic replacement of *ply* with an erythromycin resistance cassette amplified from ΔPLY2 as previously described (37). JLB12 was cultured using selective medium containing 3 µg/ml erythromycin. P. aeruginosa strains were grown at 37°C on MacConkey agar (MA) or in Luria-Bertani (LB) broth with shaking at 180 rpm. P. aeruginosa 103-29 and the isogenic protease IV (*piv*) deletion mutant have been previously characterized (38). During murine pulmonary coinfections, lung and blood samples were plated on BA containing 200 µg/ml polymyxin B to select for S. pneumoniae growth and MA without antibiotics to select for P. aeruginosa growth.

### Murine pulmonary infections.

Adult B6 mice (10 to 12 weeks old, 20 to 25 g) were anesthetized with an intraperitoneal injection of 500 µl Avertin (6.25 mg tribromoethanol dissolved in 2.5% amylene hydrate solution) and intratracheally inoculated with 50 µl PBS containing 10^6^ CFU pneumococci or 10 µg of PIV. For bacterial coinfections, mice were inoculated with 50 µl containing 10^3^ CFU of *Pseudomonas* followed by 50 µl containing 10^6^ CFU of pneumococci at 24 h after *Pseudomonas* infection. For mixed exogenous administration, mice were inoculated with 50 µl containing 10^6^ CFU of pneumococci and 10 µg of either PIV, heat-inactivated PIV, or LasB. The Chromozym PL assay (Roche) was used according to the manufacturer’s instructions to measure the proteolytic activity in fresh lung homogenates of mice administered PIV or infected with 10^6^ CFU of P. aeruginosa 103-29. Mice were monitored for survival and euthanized at 2 days postinfection. Blood was collected by retroorbital bleed or from the heart. Lungs were removed and homogenized in 2 ml PBS. Blood and lung samples were plated on selective BA or MA to enumerate CFU per milliliter recovered from each mouse. Mouse studies were approved by the University of Mississippi Medical Center Institutional Animal Care and Use Committee in accordance with National Institutes of Health laboratory animal use guidelines.

### Pneumococcal adherence to human pulmonary epithelial cells.

Adhesion assays were performed as previously described (39). Briefly, human A549 pulmonary epithelial cells that had reached at least 90% confluence in 24-well plates were washed three times with PBS and incubated with approximately 10^6^ CFU of pneumococci in each well. Pneumococci were allowed to adhere for 30 min before epithelial cells were washed three times with PBS, lifted with 0.25% trypsin-EDTA, suspended in PBS, and plated on BA to enumerate adherent pneumococcal CFU. Recovered pneumococcal CFU were adjusted by standardizing inocula to 10^6^ CFU.

### Histology of murine lungs.

At 24 h postinfection, mice were euthanized and all five lobes were fixed in modified Davidson’s fixative (20% ethanol, 6% acetic acid, 10% formaldehyde dissolved in water). Fixed tissues were processed and stained with hematoxylin and eosin (H&E) by Excalibur Pathology Inc. Stained slides were examined by a reviewer blind to the specimen source. Pneumonia was characterized by exudate of fibrin and neutrophils within the airspaces in order to determine inflammation loci (focal versus diffuse) and severity of pneumonia (severe versus mild).

### IL-22 levels in murine lungs.

Murine lungs were homogenized in PBS containing 1× Halt protease inhibitor cocktail (Thermo Scientific). Lung homogenates were centrifuged at 1,500 × *g*, and supernatants were collected for use in a sandwich enzyme-linked immunosorbent assay (ELISA). IL-22 levels in supernatants were quantified using the mouse IL-22 Ready-Set-Go! ELISA kit according to the manufacturer’s instructions.

### Statistical analysis.

Results were analyzed using the InStat program (Prism 4 software; GraphPad, San Diego, CA). The Mann-Whitney test was used to test significant differences in the means of two groups. The Kruskal-Wallis test with Dunn’s *post hoc* comparisons was used for analysis of differences in means for three or more groups. Survival curves were analyzed with a log rank test. A *P* value of <0.05 was considered statistically significant.

## References

[1] Liu L, Johnson HL, Cousens S, Perin J, Scott S, Lawn JE, Rudan I, Campbell H, Cibulskis R, Li M, Mathers C, Black RE, Child Health Epidemiology Reference Group of WHO and UNICEF 2012 Global, regional, and national causes of child mortality: an updated systematic analysis for 2010 with time trends since 2000. Lancet 379:2151–2161. doi:10.1016/S0140-6736(12)60560-1. 22579125

[2] Huang SS, Johnson KM, Ray GT, Wroe P, Lieu TA, Moore MR, Zell ER, Linder JA, Grijalva CG, Metlay JP, Finkelstein JA 2011 Healthcare utilization and cost of pneumococcal disease in the United States. Vaccine 29:3398–3412. doi:10.1016/j.vaccine.2011.02.088. 21397721

[3] Beasley V, Joshi PV, Singanayagam A, Molyneaux PL, Johnston SL, Mallia P 2012 Lung microbiology and exacerbations in COPD. Int J Chron Obstruct Pulmon Dis 7:555–569. doi:10.2147/COPD.S28286. 22969296 PMC3437812

[4] Craven DE, Steger KA 1995 Epidemiology of nosocomial pneumonia: a new perspective on an old disease. Chest 108:1S–16S. doi:10.1378/chest.108.2_Supplement.1S. 7634921

[5] Lieberman D, Schlaeffer F, Boldur I, Lieberman D, Horowitz S, Friedman MG, Leiononen M, Horovitz O, Manor E, Porath A 1996 Multiple pathogens in adult patients admitted with community-acquired pneumonia: a one year prospective study of 346 consecutive patients. Thorax 51:179–184. doi:10.1136/thx.51.2.179. 8711652 PMC473032

[6] de Roux A, Ewig S, García E, Marcos MA, Mensa J, Lode H, Torres A 2006 Mixed community-acquired pneumonia in hospitalised patients. Eur Respir J 27:795–800. doi:10.1183/09031936.06.00058605. 16585087

[7] Gutiérrez F, Masiá M, Rodríguez JC, Mirete C, Soldán B, Padilla S, Hernández I, Royo G, Martin-Hidalgo A 2005 Community-acquired pneumonia of mixed etiology: prevalence, clinical characteristics, and outcome. Eur J Clin Microbiol Infect Dis 24:377–383. doi:10.1007/s10096-005-1346-2. 15931452

[8] Okada F, Ando Y, Matsushita S, Ishii R, Nakayama T, Morikawa K, Ono A, Maeda T, Mori H 2012 Thin-section CT findings of patients with acute *Streptococcus pneumoniae* pneumonia with and without concurrent infection. Br J Radiol 85:e357–e364. doi:10.1259/bjr/18544730. 22215884 PMC3587092

[9] Dickson RP, Erb-Downward JR, Freeman CM, McCloskey L, Beck JM, Huffnagle GB, Curtis JL 2015 Spatial variation in the healthy human lung microbiome and the adapted island model of lung biogeography. Ann Am Thorac Soc 12:821–830. doi:10.1513/AnnalsATS.201501-029OC. 25803243 PMC4590020

[10] Dickson RP 2016 The microbiome and critical illness. Lancet Respir Med 4:59–72. doi:10.1016/S2213-2600(15)00427-0. 26700442 PMC4752077

[11] Dickson RP, Erb-Downward JR, Prescott HC, Martinez FJ, Curtis JL, Lama VN, Huffnagle GB 2015 Intraalveolar catecholamines and the human lung microbiome. Am J Respir Crit Care Med 192:257–259. doi:10.1164/rccm.201502-0326LE. 26177175 PMC4532827

[12] Schmidt A, Belaaouaj A, Bissinger R, Koller G, Malleret L, D’Orazio C, Facchinelli M, Schulte-Hubbert B, Molinaro A, Holst O, Hammermann J, Schniederjans M, Meyer KC, Damkiaer S, Piacentini G, Assael B, Bruce K, Häußler S, LiPuma JJ, Seelig J, Worlitzsch D, Döring G 2014 Neutrophil elastase-mediated increase in airway temperature during inflammation. J Cyst Fibros 13:623–631. doi:10.1016/j.jcf.2014.03.004. 24713593

[13] Meduri GU, Kanangat S, Stefan J, Tolley E, Schaberg D 1999 Cytokines IL-1β, IL-6, and TNF-α enhance *in vitro* growth of bacteria. Am J Respir Crit Care Med 160:961–967. doi:10.1164/ajrccm.160.3.9807080. 10471625

[14] Ishida T, Tachibana H, Ito A, Ikeda S, Furuta K, Nishiyama A, Noyama M, Tokioka F, Yoshioka H, Arita M 2015 Clinical characteristics of pneumonia in bedridden patients receiving home care: a 3-year prospective observational study. J Infect Chemother 21:587–591. doi:10.1016/j.jiac.2015.04.013. 26026661

[15] Grau I, Ardanuy C, Schulze MH, Liñares J, Pallares R 2017 Polymicrobial pneumococcal bacteraemia: a case-control study. Eur J Clin Microbiol Infect Dis 36:911–915. doi:10.1007/s10096-016-2885-4. 28054228

[16] Millares L, Ferrari R, Gallego M, Garcia-Nuñez M, Pérez-Brocal V, Espasa M, Pomares X, Monton C, Moya A, Monsó E 2014 Bronchial microbiome of severe COPD patients colonised by *Pseudomonas aeruginosa*. Eur J Clin Microbiol Infect Dis 33:1101–1111. doi:10.1007/s10096-013-2044-0. 24449346 PMC4042013

[17] Briles DE, Hollingshead SK, Paton JC, Ades EW, Novak L, van Ginkel FW, Benjamin WH 2003 Immunizations with pneumococcal surface protein A and pneumolysin are protective against pneumonia in a murine model of pulmonary infection with *Streptococcus pneumoniae*. J Infect Dis 188:339–348. doi:10.1086/376571. 12870114

[18] Guillon A, Brea D, Morello E, Tang A, Jouan Y, Ramphal R, Korkmaz B, Perez-Cruz M, Trottein F, O’Callaghan RJ, Gosset P, Si-Tahar M 2017 *Pseudomonas aeruginosa* proteolytically alters the interleukin 22-dependent lung mucosal defense. Virulence 8:810–820. doi:10.1080/21505594.2016.1253658. 27792459 PMC5626239

[19] Malloy JL, Veldhuizen RAW, Thibodeaux BA, O’Callaghan RJ, Wright JR 2005 *Pseudomonas aeruginosa* protease IV degrades surfactant proteins and inhibits surfactant host defense and biophysical functions. Am J Physiol Lung Cell Mol Physiol 288:L409–L418. doi:10.1152/ajplung.00322.2004. 15516485

[20] Sonnenberg GF, Fouser LA, Artis D 2011 Border patrol: regulation of immunity, inflammation and tissue homeostasis at barrier surfaces by IL-22. Nat Immunol 12:383–390. doi:10.1038/ni.2025. 21502992

[21] Crouch E, Wright JR 2001 Surfactant proteins A and D and pulmonary host defense. Annu Rev Physiol 63:521–554. doi:10.1146/annurev.physiol.63.1.521. 11181966

[22] Trevejo-Nunez G, Elsegeiny W, Conboy P, Chen K, Kolls JK 2016 Critical role of IL-22/IL22-RA1 signaling in pneumococcal pneumonia. J Immunol 197:1877–1883. doi:10.4049/jimmunol.1600528. 27456484 PMC4992592

[23] Jounblat R, Clark H, Eggleton P, Hawgood S, Andrew PW, Kadioglu A 2005 The role of surfactant protein D in the colonisation of the respiratory tract and onset of bacteraemia during pneumococcal pneumonia. Respir Res 6:126. doi:10.1186/1465-9921-6-126. 16255775 PMC1282592

[24] Berry AM, Ogunniyi AD, Miller DC, Paton JC 1999 Comparative virulence of *Streptococcus pneumoniae* strains with insertion-duplication, point, and deletion mutations in the pneumolysin gene. Infect Immun 67:981–985. 9916120 10.1128/iai.67.2.981-985.1999 PMC96416

[25] Berry AM, Yother J, Briles DE, Hansman D, Paton JC 1989 Reduced virulence of a defined pneumolysin-negative mutant of *Streptococcus pneumoniae*. Infect Immun 57:2037–2042. 2731982 10.1128/iai.57.7.2037-2042.1989 PMC313838

[26] Orihuela CJ, Gao G, Francis KP, Yu J, Tuomanen EI 2004 Tissue-specific contributions of pneumococcal virulence factors to pathogenesis. J Infect Dis 190:1661–1669. doi:10.1086/424596. 15478073

[27] Kadioglu A, Taylor S, Iannelli F, Pozzi G, Mitchell TJ, Andrew PW 2002 Upper and lower respiratory tract infection by *Streptococcus pneumoniae* is affected by pneumolysin deficiency and differences in capsule type. Infect Immun 70:2886–2890. doi:10.1128/IAI.70.6.2886-2890.2002. 12010976 PMC128015

[28] Caballero AR, Moreau JM, Engel LS, Marquart ME, Hill JM, O’Callaghan RJ 2001 *Pseudomonas aeruginosa* protease IV enzyme assays and comparison to other *Pseudomonas* proteases. Anal Biochem 290:330–337. doi:10.1006/abio.2001.4999. 11237336

[29] Lucas R, Czikora I, Sridhar S, Zemskov EA, Oseghale A, Circo S, Cederbaum SD, Chakraborty T, Fulton DJ, Caldwell RW, Romero MJ 2013 Arginase 1: an unexpected mediator of pulmonary capillary barrier dysfunction in models of acute lung injury. Front Immunol 4:1–7. doi:10.3389/fimmu.2013.00228. 23966993 PMC3736115

[30] Thibodeaux BA, Caballero AR, Dajcs JJ, Marquart ME, Engel LS, O’Callaghan RJ 2005 *Pseudomonas aeruginosa* protease IV: a corneal virulence factor of low immunogenicity. Ocul Immunol Inflamm 13:169–182. doi:10.1080/09273940490518937. 16019676

[31] Van Maele L, Carnoy C, Cayet D, Ivanov S, Porte R, Deruy E, Chabalgoity JA, Renauld JC, Eberl G, Benecke AG, Trottein F, Faveeuw C, Sirard JC 2014 Activation of type 3 innate lymphoid cells and interleukin 22 secretion in the lungs during *Streptococcus pneumoniae* infection. J Infect Dis 210:493–503. doi:10.1093/infdis/jiu106. 24577508

[32] Malley R, Henneke P, Morse SC, Cieslewicz MJ, Lipsitch M, Thompson CM, Kurt-Jones E, Paton JC, Wessels MR, Golenbock DT 2003 Recognition of pneumolysin by toll-like receptor 4 confers resistance to pneumococcal infection. Proc Natl Acad Sci U S A 100:1966–1971. doi:10.1073/pnas.0435928100. 12569171 PMC149942

[33] Palmer M 2001 The family of thiol-activated, cholesterol-binding cytolysins. Toxicon 39:1681–1689. doi:10.1016/S0041-0101(01)00155-6. 11595631

[34] Kadioglu A, Weiser JN, Paton JC, Andrew PW 2008 The role of *Streptococcus pneumoniae* virulence factors in host respiratory colonization and disease. Nat Rev Microbiol 6:288–301. doi:10.1038/nrmicro1871. 18340341

[35] Traidej M, Marquart ME, Caballero AR, Thibodeaux BA, O’Callaghan RJ 2003 Identification of the active site residues of *Pseudomonas aeruginosa* protease IV: importance of enzyme activity in autoprocessing and activation. J Biol Chem 278:2549–2553. doi:10.1074/jbc.M208973200. 12419815

[36] Andersson B, Dahmén J, Frejd T, Leffler H, Magnusson G, Noori G, Edén CS 1983 Identifiation of an active disaccharide unit of a glycoconjugate receptor for pneumococci attaching to human pharyngeal epithelial cells. J Exp Med 158:559–570. doi:10.1084/jem.158.2.559. 6886624 PMC2187347

[37] Keller LE, Bradshaw JL, Pipkins H, McDaniel LS 2016 Surface proteins and pneumolysin of encapsulated and nonencapsulated *Streptococcus pneumoniae* mediate virulence in a chinchilla model of otitis media. Front Cell Infect Microbiol 6:1–11. doi:10.3389/fcimb.2016.00055. 27242973 PMC4870244

[38] Caballero A, Thibodeaux B, Marquart M, Traidej M, O’Callaghan R 2004 *Pseudomonas* keratitis: protease IV gene conservation, distribution, and production relative to virulence and other *Pseudomonas* proteases. Invest Ophthalmol Vis Sci 45:522–530. doi:10.1167/iovs.03-1050. 14744894

[39] Keller LE, Jones CV, Thornton JA, Sanders ME, Swiatlo E, Nahm MH, Park IH, McDaniel LS 2013 PspK of *Streptococcus pneumoniae* increases adherence to epithelial cells and enhances nasopharyngeal colonization. Infect Immun 81:173–181. doi:10.1128/IAI.00755-12. 23115034 PMC3536125

